# Varenicline as a smoking cessation aid in a Greek population: a subanalysis of an observational study

**DOI:** 10.1186/1617-9625-10-1

**Published:** 2012-02-02

**Authors:** Christina Gratziou, Konstantinos Gourgoulianis, Paraskevi Argyropoulou Pataka, Georgia D Sykara, Michael Messig, Sunil Raju

**Affiliations:** 1 Evgenidio Hospital, Medical School, University of Athens, Athens, Greece; 2 Pulmonary Department, Medical School, University of Thessaly, Thessaly, Greece; 3 Aristotle University of Thessaloniki, Thessaloniki, Greece; 4 Pfizer, Athens, Greece; 5 Pfizer Inc, New York, New York, USA; 6 Pfizer Ltd, Tadworth, Surrey, UK

**Keywords:** smoking cessation, Greece, varenicline, real world, observational

## Abstract

**Background:**

Greece has the highest proportion of smokers in the European Union with 42% of Greeks admitting that they smoke, based on a 2009 survey. This post-hoc analysis of a prospective, observational study evaluated the effectiveness and safety profile of the smoking cessation aid varenicline, as well as potential predictors of quit success in a Greek population.

**Methods:**

Participants were prescribed varenicline according to the recommendations of the European Summary of Product Characteristics (1 mg twice daily). The 7-day point prevalence of abstinence at Week 12 was determined based on verbal reporting using a nicotine use inventory. Abstinence was confirmed by carbon monoxide measurements of exhaled air at the last visit of the study. The safety profile of varenicline was also assessed.

**Results:**

At baseline, the Greek subsample (n = 196) had a mean age of 42.6 years, with 54.6% of them being men. Participants had a smoking history of 23.5 years and a Fagerström Test for Nicotine Dependence total score of 6.6. After 12 weeks of varenicline therapy, 70.4% (95% CI, 64.0-76.7) of all participants had quit smoking. This increased to 86.2% among participants who had taken the study medication for 80% of the planned number of treatment days. Age was a significant predictor of quit success. The most frequently observed treatment-emergent adverse event was nausea, occurring in 13.3% of participants.

**Conclusions:**

In this 'real-world' observational study, 70.4% of Greek smokers successfully quit smoking after 12 weeks of varenicline therapy, providing support that varenicline is an effective smoking cessation medication. Further studies with longer follow-up are warranted.

**Trial Registration:**

ClinicalTrials.gov: NCT00669240

## Introduction

Tobacco use accounts for over half a million deaths each year in the European Union (EU), with comprehensive public health policies currently being developed to curb this epidemic in member countries [1]. Smoking cessation is a key element of these EU-wide policies. However, the success rate of quit attempts without pharmacological assistance is low: only 3-5% of self-quitters remain abstinent after 6-12 months following a quit attempt [2]. Current guidelines recommend the use of smoking cessation medications combined with behavioural support to address tobacco dependency and improve abstinence rates following a quit attempt [3].

Varenicline is among the first-line smoking cessation aids recommended in the Clinical Practice Guidelines by the United States Public Health Service [3]. A partial agonist of the nicotinic α4β2 receptor, it is believed to reduce nicotine withdrawal symptoms by maintaining moderate dopamine levels and diminishing smoking satisfaction [4]. Its efficacy and safety has been demonstrated in several randomized, double-blind, placebo-controlled clinical trials [5-10]. Furthermore, a meta-analysis of various pharmacological interventions for smoking cessation found varenicline to be superior to bupropion and placebo as a smoking cessation aid [11].

In post-marketing surveillance, there have been reports of psychiatric adverse events. However, a pooled quantitative safety analysis of 10 randomized, double-blind, placebo-controlled clinical trials of varenicline in participants without psychiatric disease found no significant association between psychiatric adverse events and varenicline use, with the exception of sleep disorders and disturbances [12].

Stringent inclusion and exclusion criteria applied in randomized, controlled trials mean that participants with chronic comorbidities or on medication are excluded. The value of observational studies lies in the heterogeneity of participants who are included, making the findings of observational studies more representative of the general population compared with randomized, controlled trials.

The CHampix Observational Investigation in the Cessation of Smoking (CHOICES) study investigated varenicline as a smoking cessation aid in routine clinical practice in four European countries (Belgium, Greece, Hungary and Slovenia): a self-reported point prevalence of abstinence of 64.6% after 12 weeks of treatment with varenicline was reported [13].

Greece has the highest proportion of smokers in the EU and among member countries of the Organisation for Economic Cooperation and Development: in a 2009 survey on tobacco use in European countries, 42% of Greek respondents admitted that they smoke [1,14]. Smoking-related diseases kill approximately 20,000 people a year in Greece, costing the country an annual €2.14 billion (£1.8 billion), according to a statement by the Greek Health Ministry in 2009 [15,16]. This subanalysis of the CHOICES study was conducted to evaluate the real-life effectiveness and safety profile of varenicline in a Greek population.

## Methods

### Study design and participants

The CHOICES study was a 12-week, prospective, observational, non-comparative trial with 551 participants conducted in four European countries (Belgium, Greece, Hungary and Slovenia) between 21 November 2007 and 3 August 2009 [13]. In this post-hoc analysis, only results from the 196 participants recruited at 10 study centres in Greece are presented.

The final protocol, any amendments, and informed consent documentation were reviewed and approved by the Independent Ethics Committee at each of the investigational centers participating in the study. This study was conducted in compliance with the ethical principles originating in or derived from the Declaration of Helsinki and in compliance with the Independent Ethics Committees, informed consent regulations, and generally accepted research practices such as the Good Pharmacoepidemiology Practices issued by the International Society for Pharmacoepidemiology.

Participants were eligible for inclusion in the study if they were adults of legal age who smoked regularly, with the main tobacco product smoked being cigarettes, who were willing to make an attempt to stop smoking, were deemed to be suitable for varenicline treatment and motivated to quit in the judgement of their supervising physician.

Study visits occurred at enrolment and Week 12. Interim visits were permitted if they were part of a study site's normal practice for smoking cessation.

At enrollment, all demographic data (including age and body mass index [BMI; defined as body weight measured in kilograms divided by the square of height measured in meters]), smoking history, nicotine dependence and smoking-related illnesses were recorded.

Varenicline was administered in accordance with the European Summary of Product Characteristics (SmPC) [17] stating that: (i) varenicline treatment should commence 1-2 weeks before the quit date; (ii) for the first 3 days, varenicline should be administered as 0.5 mg once daily, then uptitrated to 0.5 mg twice daily on Days 4-7; from Day 8 to the end of the 12-week treatment phase varenicline should be administered as 1 mg twice daily. Behavioural support was available to participants according to the site's usual practice (e.g., counselling: reinforcement and discussion about withdrawal problems in follow-up visits [visit time 15-30 minutes]).

### Prior and concomitant medications and comorbidities

There were no restrictions on prior (i.e., before start of study treatment) and concomitant (i.e., during the study treatment period) medications or comorbidities apart from the usual prescribing information in the SmPC [17].

### Measurements

#### Smoking cessation

The use of nicotine during the 7-day period between Weeks 11 and 12 (7-day point prevalence of abstinence [7-day PPA]) was recorded based on verbal reporting using a nicotine use inventory (NUI) to determine the smoking status of the participant. The NUI consisted of the following two questions: whether the person had smoked "any cigarettes (even a puff)" and "any other tobacco products (eg, pipe, cigars, snuff, chew)".

#### Fagerström Test of Nicotine Dependence

Nicotine dependence was assessed at enrolment using the Fagerström Test of Nicotine Dependence (FTND), which consists of six questions [18]. Question 1 of the FTND read as follows: "How soon after you wake up do you smoke your first cigarette?" A total score was calculated as a sum of the six questions, with lower scores indicating lower dependence on nicotine: 0-2, very low dependence; 3-4, low dependence; 5, medium dependence; 6-7, high dependence; and 8-10, very high dependence. Exhaled carbon monoxide was measured at each visit. Abstinence was confirmed by carbon monoxide measurements of exhaled air at the last visit of the study.

### Safety evaluations

To assess safety and tolerability of varenicline, investigators recorded all observed or participant-reported adverse events, including their opinion regarding the adverse event intensity (mild [did not interfere with participant's usual function], moderate [interferes to some extent with participant's usual function], or severe [interferes significantly with participant's usual function]), as well as the investigators' expert medical opinions on the relationship of the adverse events to the study treatment. These adverse events were coded using the *Medical Dictionary for Regulatory Activities *(MedDRA), version 12. For all adverse events, the investigators determined both the adverse event outcome, as well as whether any specific adverse events met the criteria for classification as a serious adverse event. A serious adverse event was defined as any adverse event that was life-threatening; caused death; resulted in new, or prolongation of existing, in-patient hospitalisation; led to a persistent or significant disability/incapacity; resulted in a congenital anomaly/birth defect; or if they required medical or surgical intervention to prevent one of these outcomes listed. Moreover, serious adverse events could also include any adverse event that the sponsor or investigators considered serious enough to be classified as such. Safety data were collected and assessed using the same procedures and standards as in previous clinical trials of varenicline.

### Statistical analysis

The analysis included all enrolled participants in Greece who had received at least one dose of study medication. Descriptive summaries were provided as counts and percentages for categorical variables and as means and standard deviations for continuous variables. Exact Clopper-Pearson 95% confidence limits for the abstinence rates were determined based on the binomial distribution.

7-day PPA by baseline age, baseline FTND total score and by baseline FTND Question 1 response (ie, time to first cigarette on waking) were analyzed using univariate logistic regression models to compare groups. The analyses were performed using SAS version 9.2.

## Results

### Demographics and smoking history

In total, 196 Greek smokers received varenicline in this study. Their mean age was 42.6 years, with 54.6% of them being men (Table 1). The mean duration of smoking was 23.5 years, with a mean number of 29.7 cigarettes being smoked per day. The total FTND score was 6.6 for this population, indicating a high dependence on nicotine, with most participants (42.1%) smoking between 21 and 30 cigarettes per day (Table 1).

**Table 1:** Baseline demographics

	Men (n = 107)	Women (n = 85)	Total (n = 196)*
Characteristics			

Age, years, mean (range)	43.7 (22-67)	41.3 (19-67)	42.6 (19-67)
BMI, kg/m^2 ^(SD)	28.3 (4.6)	24.0 (4.2)	26.3 (4.9)

Medical history related to smoking, mean (SD)			

Total number of years smoked	25.1 (10.8)	21.4 (8.4)	23.5 (10.0)
Mean number of cigarettes per day	32.5 (15.5)	26.2 (11.9)	29.7 (14.3)
Severity of smoking, n (%)			
≤ 10 cigarettes/day	0 (0.0)	6 (7.0)	6 (3.1)
11-20 cigarettes/day	23 (21.4)	26 (30.5)	49 (25.5)
21-30 cigarettes/day	45 (42.0)	36 (42.3)	81 (42.1)
> 30 cigarettes/day	39 (36.4)	17 (20.0)	56 (29.1)
FTND total score	6.7 (2.2)	6.4 (2.3)	6.6 (2.2)
FTND question 1 response, n (%)			
< 5 mins	45 (42.0)	41 (48.2)	86 (44.7)
5-30 mins	40 (37.3)	26 (30.5)	66 (34.3)
31-60 mins	13 (12.1)	15 (17.6)	28 (14.5)
> 60 mins	9 (8.4)	3 (3.5)	12 (6.2)

BMI, body mass index; SD, standard deviation; FTND, Fagerström Test of Nicotine Dependence

* n = 4 with unspecified gender; n/a, not available.

### Prior and concomitant medications and comorbidities

The most frequently used prior and concomitant medications were aspirin (9 out of 53 [17.0%]), levothyroxine sodium (8 out of 53 [15.1%]) and atorvastatin (6 out of 53 [11.3%]). The most frequently observed comorbidities were hypertension (16.8%), chronic obstructive pulmonary disease (8.2%) and depression (7.1%) (Table 2).

**Table 2:** Comorbidities occurring in ≥2% of participants

Hypertension, n (%)	33 (16.8)
Chronic obstructive pulmonary disease, n (%)	16 (8.2)
Depression, n (%)	14 (7.1)
Diabetes mellitus, n (%)	11 (5.6)
Hypothyroidism, n (%)	8 (4.1)
Asthma, n (%)	8 (4.1)
Epilepsy, n (%)	6 (3.1)
Migraine, n (%)	4 (2.0)

### Compliance and duration of treatment exposure

Overall, 40 (20.4%) Greek participants in this subanalysis discontinued from the study: 16 (8.2%) due to study drug (adverse events, 5 [2.6%]; and lack of efficacy, 11 [5.6%]) and 24 (12.2%) for reasons that were unrelated to study drug (lost to follow-up, 6 [3.1%]; subject no longer willing to participate in this study, 16 [8.2%]; other, 2 [1.0%]). The median duration of treatment was 84.0 days, with 39.7% of participants taking varenicline for 61-90 days.

### Outcomes

**Figure 1:**
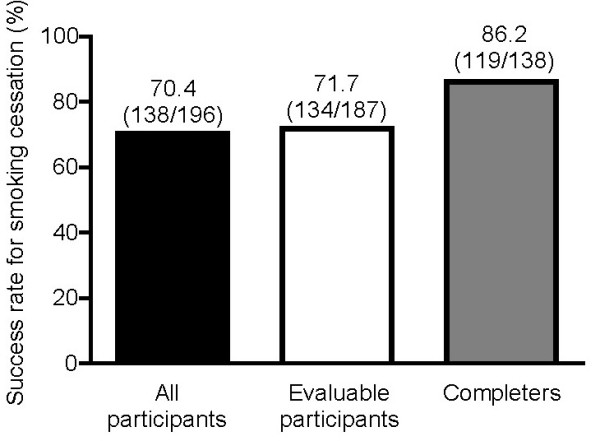
**Success rates for smoking cessation in the self-reported 7-day period between Weeks 11 and 12 (7-day point prevalence of abstinence)**. All participants, all enrolled participants who had received at least 1 dose of study medication (either morning, evening or both of the planned twice-daily doses); evaluable participants, participants who took at least 14 days of study medication in the first 21 days (a day of study medication being defined as a calendar day during which the participant received any dose of study medication); completers, participants with at least 80% of treatment compliance defined as having any dose of study medication for 80% of the planned number of treatment days.

**Figure 2:**
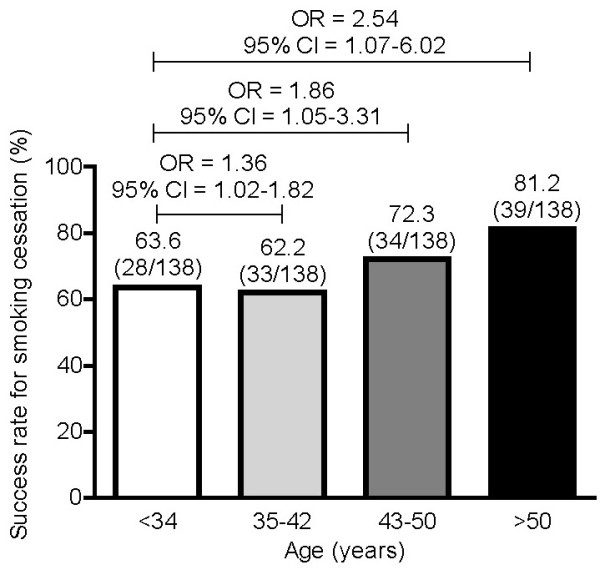
**Self-reported 7-day point prevalence of abstinence at Week 12 in Greek participants of the CHOICES study by baseline age**. Age was unknown for 4 participants. p = 0.035 for overall age group differences based on univariate logistic regression model.

By the end of the 12-week treatment period, 70.4% (95% confidence interval [C.I.] 64.0-76.7) of all participants had quit smoking (Figure 1). The rate of abstinence increased with better compliance. We considered as 'completers' those participants who had a good treatment compliance defined as taking study medication for 80% of the planned number of treatment days. A total of 119 of 138 (86.2%) smokers who were characterized as completers had quit smoking by Week 12 (Figure 1). Furthermore, abstinence increased significantly with increasing age (Figure 2).

**Figure 3:**
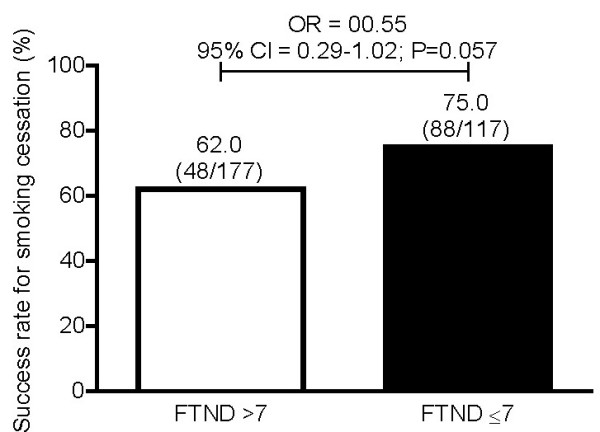
**Self-reported 7-day point prevalence of abstinence at Week 12 in Greek participants of the CHOICES study by baseline nicotine dependence as assessed by the FTND total score**. The FTND total score was not available for 2 participants. These 2 individuals were excluded from this analysis. Based on univariate logistic regression model.

When the relationship between successful smoking cessation and the FTND total score were investigated, it was found that in participants with a total FTND score of > 7, 62.0% had successfully quit smoking at Week 12 (Figure 3). In participants with a lower dependence on nicotine (ie, an FTND score of ≤ 7), the quit rate was even higher (ie, 75.0%) (Figure 3). However, there was no statistically significant difference between these quit rates (p = 0.057), therefore the FTND total score was not predictive of successful smoking cessation in this analysis although the results approached significance. Likewise, there was no statistically significant relationship between successful smoking cessation and the severity of smoking at baseline (p = 0.399) and between abstinence and gender (p = 0.567).

**Figure 4:**
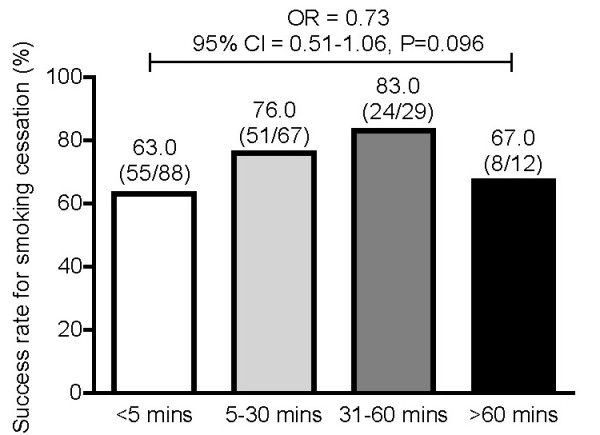
**Self-reported 7-day point prevalence of abstinence at Week 12 in Greek participants of the CHOICES study by baseline FTND Question 1 response ("How soon after you wake up do you smoke your first cigarette?")**. OR = Relative change in odds for shift to an adjacent category going from longer times to shorter times based on univariate logistic regression model.

The relationship between successful smoking cessation and Question 1 of the FTND ("How soon after you wake up do you smoke your first cigarette?") was also investigated using logistic regression models. There was a trend, albeit not statistically significant, for decreased odds of success with decreasing time to the first cigarette within 60 minutes after waking up (Figure 4).

### Safety/tolerability

The most frequently observed treatment-emergent adverse events (all-causality) were nausea (13.3%), insomnia (4.6%) and upper abdominal pain (4.1%), mostly of mild to moderate intensity (Table 3). These adverse events are already listed in the SmPC [17]. The most frequent psychiatric disorders were insomnia (4.6%), abnormal dreams (3.6%) and nervousness (1.0%). These symptoms were controlled within 10 days in most of the participants. No serious adverse events were observed.

**Table 3:** Incidence of treatment-emergent adverse events (all causality) in ≥ 1 participants by intensity

	Total population (N = 196)			
	**Total** **n (%)**	**Mild Intensity** **n (%)**	**Moderate Intensity** **n (%)**	**Severe Intensity** **n (%)**

Nausea	26 (13.3)	17 (8.7)	7 (3.6)	2 (1.0)
Insomnia	9 (4.6)	6 (3.1)	2 (1.0)	1 (0.5)
Abdominal pain, upper	8 (4.1)	4 (2.0)	1 (0.5)	3 (1.5)
Abnormal dreams	7 (3.6)	4 (2.0)	3 (1.5)	0 (0.0)
Dry mouth	4 (2.0)	3 (1.5)	1 (0.5)	0 (0.0)
Constipation	3 (1.5)	0 (0.0)	2 (1.0)	1 (0.5)
Nervousness	2 (1.0)	2 (1.0)	0 (0.0)	0 (0.0)
Migraine	2 (1.0)	1 (0.5)	1 (0.5)	0 (0.0)
Somnolence	2 (1.0)	2 (1.0)	0 (0.0)	0 (0.0)
Gastrointestinal disorder	2 (1.0)	2 (1.0)	0 (0.0)	0 (0.0)
Vomiting	2 (1.0)	2 (1.0)	0 (0.0)	0 (0.0)

## Discussion

The findings of this subanalysis of CHOICES indicate that a high smoking abstinence was achieved in the Greek population of this study, despite participants' heavy dependence on nicotine. This study has shown that treatment with varenicline as provided by a smoking cessation clinic outside a clinical trial environment can be very effective as a smoking cessation treatment.

Overall, 70.4% of Greek participants successfully quit smoking at the end of a 12-week course of varenicline, as assessed by self-reported 7-day PPA as a measure of abstinence. Neither the total FTND score nor FTND Question 1 response (ie, time to first cigarette on waking) significantly predicted a successful 7-day PPA at Week 12. The most frequently observed adverse events were nausea (13.3%), insomnia (4.6%) and upper abdominal pain (4.1%), mostly of mild to moderate severity.

The efficacy and tolerability of varenicline has been shown in various placebo-controlled, double-blind, randomized clinical trials [11,12]. Stringent inclusion and exclusion criteria applied in these trials meant that participants with chronic comorbidities and medication were excluded. The value of observational studies, such as CHOICES, lies in the heterogeneity of participants who are enrolled, making the findings more representative of the general population. For example, in the analysis presented here, a high proportion of participants had a medical history of hypertension, chronic obstructive pulmonary disease or depression and had effective treatment for smoking cessation with varenicline. These findings support varenicline use in populations with these comorbidities. This has also been shown in double-blind clinical trials [10].

In the main CHOICES study [13] the 7-day PPA by Week 12 was 64.6% (95% C.I. 60.1-68.3), while 61.1% was reported for a subanalysis of the Belgian population enrolled in the CHOICES study[19]. This compares with a 7-day PPA of 70.4% (95% C.I. 64.0-76.7) for the Greek population at Week 12 which is reported here. The higher rate of abstinence in our population may be due to a number of factors including differences between the Greek study centres versus the other countries in the CHOICES trial (Belgium, Hungary and Slovenia), such as, differences in behavioural support and/or reimbursement. Although these factors were not compared in the CHOICES trial and a further trial would be required in order to evaluate the impact of any differences on outcomes, it is important to note that in Greece this observational study was carried out in respiratory departments and smokers were treated and monitored by the same medical doctor throughout the trial. Doctors had received training in smoking cessation strategies and were voluntarily referred to the smoking cessation clinics, where behavioural support was provided. High abstinence rates have been previously reported in these clinics using other smoking cessation treatments, such as behavioural support, nicotine replacement products and bupropion SR, in Greek smokers [20,21].

It is also important to note that at the time the CHOICES study was conducted, there was no reimbursement policy for smoking cessation medications (including varenicline) in Greece, although there are current proposals to partially reimburse Greek smokers for smoking cessation medications in the future.

One additional important finding of the study is the higher rate of abstinence with better compliance. We considered as completers those participants who had good treatment compliance, defined as taking study medication for 80% of the planned number of treatment days (more than 8 weeks). 86% of smokers who were completers had quit smoking by Week 12. This shows the greater effectiveness of varenicline treatment when it is taken for more than a month, and indicates that good compliance to treatment is important and that treatment for more than 8 weeks is associated with higher success rates. Close follow-up by specialized smoking cessation clinics increases the compliance to treatment and further the abstinence rates, as other published studies in Greek smokers have also reported [20,21].

In the Belgian subanalysis of CHOICES, the FTND total score was not predictive of successful smoking cessation at Week 12 (P = 0.12; odds ratio [O.R.] 0.65 [95% C.I. 0.38-1.11]), [22] similar to what is reported here for the Greek population (P = 0.057; O.R. 0.55 [95% C.I. 0.29-1.02]). The latter was anticipated, as traditional measures of nicotine dependence, such as the FTND, are not consistently predictive of successful smoking cessation, as reported in the literature [23].

Time to first cigarette on waking was predictive of the 7-day PPA at Week 12 in the Belgian subpopulation [22]. Although there was a similar trend in the Greek subpopulation, this was not statistically significant (O.R. 0.73, 95% C.I. 0.51-1.06, P = 0.096). This may be so because the majority of the Greek smokers in this study belong to the first two categories, lighting up their first cigarette within the first 30 minutes. In a previously published study in Greek smokers, multivariate regression analysis indicated that the time to first cigarette on waking, and use of bupropion, independently predicted abstinence [24].

In a recent observational study based on records stored in The Health Improvement Network (THIN) database in the United Kingdom, the 7-day PPA 6 months after the initiation of varenicline therapy was 49.5%, based on self-reporting [25]. A longer follow-up of the participants of the CHOICES study would be of interest and may give comparable results. A strong and positive message of this subanalysis of CHOICES is the high effectiveness of varenicline treatment in Greek smokers. Unfortunately, in Greece, the smoking ban laws are not implemented broadly in public places, and there has been no substantial media campaign supporting smoking bans [26].

## Conclusions

Despite the heavy smoking history of the Greek participants in CHOICES and negative public attitudes in Greece with regard to the indoor public smoking ban, 70% of Greek smokers successfully quit smoking after 12 weeks of varenicline therapy in this 'real-world' observational study, providing further support that varenicline is an effective smoking cessation medication. This suggests that abstinence is not related to the nicotine dependence or smoking history of a population. Personal motivation, intensive support and close follow-up by specialized smoking cessation clinics may increase patient compliance and abstinence rates. It should be noted that this subanalysis is only exploratory in nature, as the original CHOICES study was not powered to determine differences between subgroups. The long-term effectiveness of varenicline based on compliance and treatment duration and the relapse rate of Greek smokers would be interesting objectives of future studies. Thus, further studies with longer follow-up are warranted.

## List of abbreviations

CHOICES: CHampix Observational Investigation in the Cessation of Smoking study; CI: confidence interval; EU: European Union; FTND: Fagerström Test for Nicotine Dependence; MedDRA: Medical Dictionary for Regulatory Activities; NUI: Nicotine Use Inventory; OR: odds ratio; PPA: point prevalence of abstinence; SmPC: Summary of Product Characteristics.

## Competing interests

CG and PAP declare that they have no financial competing interests. KG declares a financial competing interest from the research committee of the University of Thessaly. GS, MM and RS declare financial competing interests as employees of and stock holders of Pfizer, the study sponsor. No authors declare a non-financial competing interest.

## Authors' contributions

CG was involved in the acquisition, analysis and interpretation of data; and in drafting and critically revising the manuscript. KG was involved in study conception and design; in the acquisition, analysis and interpretation of data; and in drafting and critically revising the manuscript. PAP was involved in study conception and design; and in drafting and critically revising the manuscript. GS was involved in interpretation of data and in drafting and critically revising the manuscript. MM was involved in the acquisition, analysis and interpretation of data; and in drafting and critically revising the manuscript. SR was involved in study conception and design; in the acquisition, analysis and interpretation of data; and in drafting and critically revising the manuscript. All authors read and approved the final manuscript.

## Author information

CG is Associate Professor of Pulmonary and Critical Care Medicine at Athens University Medical School; Head of Asthma Centre and Smoking Cessation Centre at Evgenidio Hospital; and Coordinator of the Working Group on Smoking Cessation of the Hellenic Thoracic Society. KG is Professor of Respiratory Medicine at the University of Thessaly. PAP is Professor of Respirology at the Aristotle University of Thessaloniki and Director of Respiratory Failure Unit at the Aristotle University of Thessaloniki. GDS (PhD), is a Medical and Scientific Relations Manager at Pfizer Hellas A.E., Greece. MM (PhD), is a Biostatistician at Pfizer Inc., New York, USA. SR (MBBS, MRCA, BSc) is a Medical Director at Pfizer Ltd, UK.
